# IgG kappa multiple myeloma with isolated central nervous system relapse

**DOI:** 10.1002/jha2.469

**Published:** 2022-05-23

**Authors:** Ke Xu, Lucy Kamuriwo, Claire Anderson, Parag Jasani

**Affiliations:** ^1^ Department of Haematology University College London Hospitals NHS Foundation Trust University College London London UK; ^2^ Specialist Integrated Haematology Malignancy Diagnostic Service, Health Services Laboratories University College London Hospitals NHS Foundation Trust University College London London UK; ^3^ Department of Haematology Barnet and Chase Farm Hospital NHS Trust London UK

1

A 76‐year‐old male presented with acute renal failure and cast nephropathy was diagnosed with IgG kappa multiple myeloma (MM). His paraprotein was 40 g/L, kappa‐free light chain level was 17,058 mg/L, kappa: lambda light chain ratio was 1445. An automated full blood count showed hemoglobin 90 g/L, white blood cells 2.96 × 10^9^/L, neutrophil 1.7 × 10^9^/L, and platelet 179 × 10^9^/L. Bone marrow trephine sample was hypercellular with nearly 100% plasma cells, which were positive for CD138, CD56, and cyclin D1, and showed kappa light chain restriction. Fluorescence in situ hybridization analysis with dual color/dual probes for immunoglobulin heavy chain (IGH) and *MYC* genes (Cytocell) and whole genome screening using 8 × 60K oligonucleotide arrays (Agilent) performed on the CD138 enriched cells (CD138 microbeads Miltenyi Biotech) from the liquid bone marrow sample showed no evidence of IGH rearrangement, *MYC* rearrangement, 1q gain, 1p loss, 17p loss, or any clinically significant imbalance.

He was treated with eight cycles of bortezomib, cyclophosphamide, and dexamethasone (VCD) to very good partial response. He relapsed 30 months after diagnosis with paraprotein of 36 g/L, kappa light chains of 10,000 mg/L, and creatinine of 498 μmol/L. He was treated with eight cycles of bortezomib, thalidomide, and dexamethasone (VTD) to paraprotein undetectable and kappa light chain 29 mg/L. Seven months post completion of VTD (4 years after myeloma diagnosis), he presented with abnormal gait, falls, and a left‐sided facial droop. His paraprotein was 2 g/L and serum‐free kappa light chain was 81 mg/L. Magnetic resonance imaging (MRI) head and whole spine showed multifocal diseases in spinal canal and intracranially, involving the cranial nerves most prominent the fifth, seventh, eighth, and hypoglossal nerve on the left, anterior surface of the inferior pons medulla (Figure 1A). There were multiple lesions in the cervical spine as well as in the thoracic spine (Figure 1B). Computerized tomography (CT) chest abdomen pelvis did not show the evidence of other new diseases. Plasma cells were detected in cerebral spinal fluid (CSF) (Figure 1C, May–Grünwald–Giemsa stain x100 objective). He was treated with pomalidomide and dexamethasone. Weekly cytarabine intrathecal (IT) treatment was given until the clearance of plasma cells from CSF samples for three times. Four months later, he achieved the complete remission on MRI head and whole spine with undetectable paraprotein and normal light chain ratio. Serial MRI scans confirmed ongoing remission. A year later, he relapsed again with worsening mobility and reappearance of myeloma cells in CSF. By flow cytometry, these cells were positive for CD138, CD38, CD56, CD117, and negative for CD19. MRI head showed leptomeningeal disease with a new 2.2 cm frontal lobe lesion. His paraprotein remained undetectable and kappa light chain remained at below 100 mg/L. He continued systemic pomalidomide and dexamethasone chemotherapy and received palliative whole brain radiotherapy 20 Gy in 5 fractions for symptom control. His disease continued progressing and he passed away 3 months later (5.5 years after myeloma diagnosis).

**FIGURE 1:**
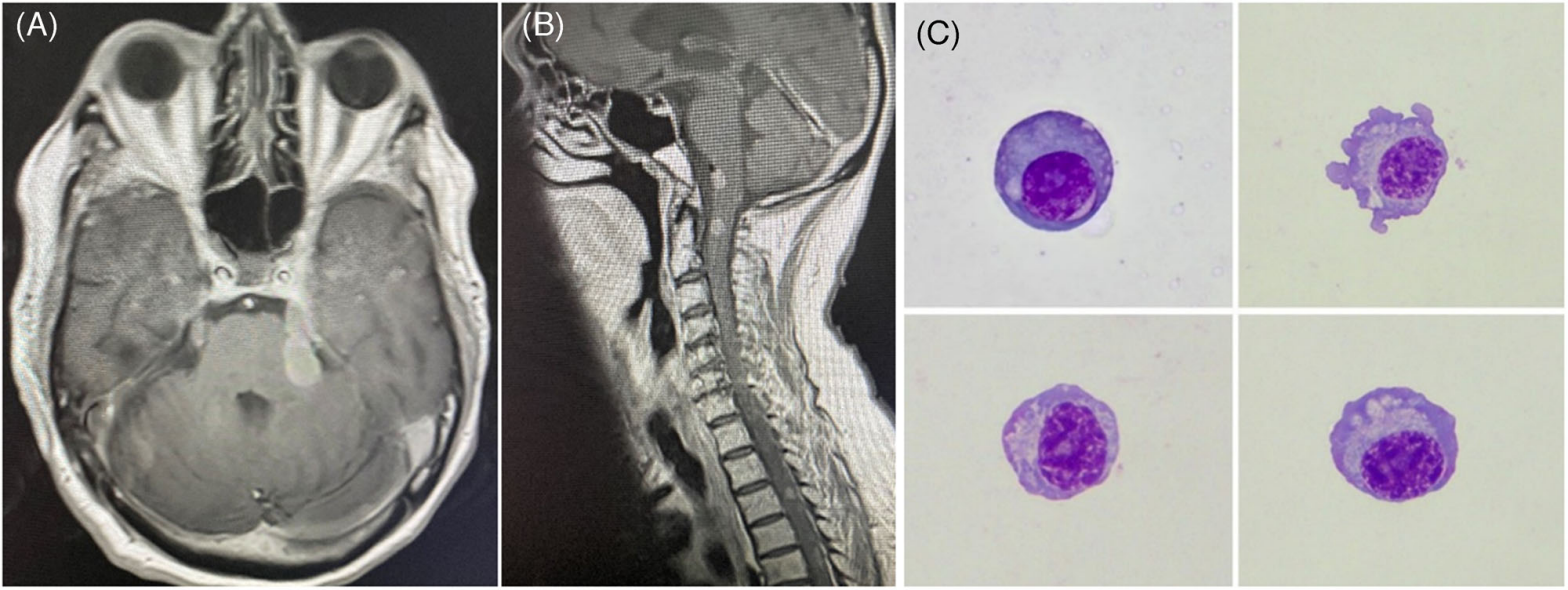
(A) MRI head; (B) MRI head and whole spine; (C) CSF (May‐Grünwald‐Giemsa stain x100 objective)

Here, we reported a myeloma patient with isolated CNS relapse, who achieved 1 year of remission with combination of intrathecal chemotherapy and systemic pomalidomide and dexamethasone treatment, but sadly passed away 4 months after second CNS relapse.

CNS MM is a rare form of extramedullary disease, but carries a very poor prognosis. The optimal approach to treatment of CNS MM is not currently known. The current approach includes systemic therapy with agents known to cross the blood brain barrier, intrathecal therapy, and CNS irradiation. Innovative approach to treatment is urgently needed.

## FUNDING

The authors received no specific funding for this work.

## CONFLICTS OF INTEREST

The authors declare they have no conflicts of interest.

## PATIENT CONSENT STATEMENT

No written or verbal consent obtained from patient. Patient has passed away.

